# Interpreting the Dependence of Mutation Rates on Age and Time

**DOI:** 10.1371/journal.pbio.1002355

**Published:** 2016-01-13

**Authors:** Ziyue Gao, Minyoung J. Wyman, Guy Sella, Molly Przeworski

**Affiliations:** 1 Committee on Genetics, Genomics and Systems Biology, University of Chicago, Chicago, Illinois, United States of America; 2 Department of Biological Sciences, Columbia University, New York, New York, United States of America; 3 Department of Systems Biology, Columbia University, New York, New York, United States of America; Institute of Science and Technology Austria (IST Austria), AUSTRIA

## Abstract

Mutations can originate from the chance misincorporation of nucleotides during DNA replication or from DNA lesions that arise between replication cycles and are not repaired correctly. We introduce a model that relates the source of mutations to their accumulation with cell divisions, providing a framework for understanding how mutation rates depend on sex, age, and cell division rate. We show that the accrual of mutations should track cell divisions not only when mutations are replicative in origin but also when they are non-replicative and repaired efficiently. One implication is that observations from diverse fields that to date have been interpreted as pointing to a replicative origin of most mutations could instead reflect the accumulation of mutations arising from endogenous reactions or exogenous mutagens. We further find that only mutations that arise from inefficiently repaired lesions will accrue according to absolute time; thus, unless life history traits co-vary, the phylogenetic “molecular clock” should not be expected to run steadily across species.

## Abstract 2

Modeling how the source of mutations relates to their rate of accumulation with age, sex, and number of cell divisions helps to explain perplexing observations about germline and somatic mutations.

## Author Summary

We relate how mutations arise to how they accumulate in different sexes, with age and with cell division. This model provides a single framework within which to interpret emerging results from evolutionary biology, human genetics, and cancer genetics. We show that the accrual of mutations should track cell divisions not only when mutations originate during DNA replication but also when they arise through non-replicative mechanisms and are repaired efficiently. This realization means that previous observations of correlations between mutation and cell division rates actually provide little support to the commonly held belief that most germline and somatic mutations arise from replication errors. We further find that only mutations that arise from inefficiently repaired lesions will accrue according to absolute time; thus, without covariation in life history traits, the phylogenetic “molecular clock” should not be expected to run at constant rates across species.

## Introduction

**Fig 1:**
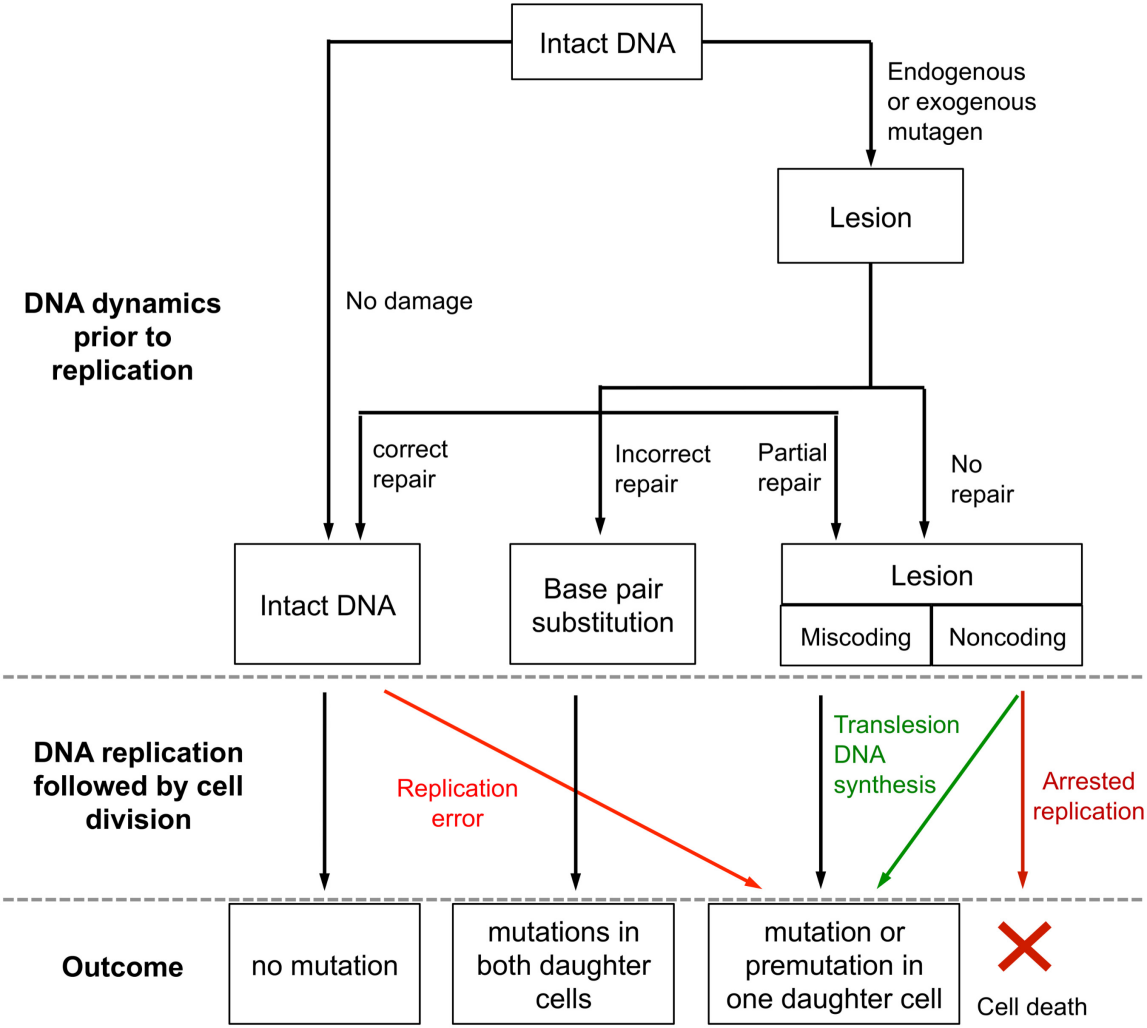
An overview of the mutagenesis process, which involves DNA damage, repair, and replication (adapted from [5]). Explanation of terms: **Lesion**: chemically altered base; **noncoding lesion**: lesion that cannot pair properly with any regular DNA bases; **miscoding lesion**: lesion that pairs with regular DNA bases that differ from the original one; **correct repair**: repair that completely reverses the lesion to the original state; **incorrect repair**: repair that recognizes the mismatch caused by lesion but alters the undamaged base by mistake; **partial repair**: incomplete repair that leads to abasic sites or other base alterations; **replication error**: misincorporation of nucleotide in the newly synthesized strand despite intact template; **translesion DNA synthesis**: damage tolerance mechanism that allows the DNA replication to bypass lesions and is often mutagenic; **point mutation**: base pair substitution; **premutation**: a base pair at which a lesion is present on one strand and the base on the other strand is substituted, as a result of DNA synthesis from incorrect template information.

Because mutations are the ultimate source of all genetic variation, deleterious and advantageous, mutagenesis has been of central interest even before the discovery of DNA as the genetic material (e.g., [1]), and developing a model of mutational heterogeneity along the genome is a major focus of current disease mapping studies [2,3]. From many decades of research into mechanisms of DNA replication, damage, and repair, we know that mutations can arise from errors during replication, such as the incorporation of a non-complementary nucleotide opposite an intact template nucleotide during DNA synthesis [4], or from DNA damage caused by exogenous mutagens or endogenous reactions at any time during normal growth of a cell (Fig 1). If uncorrected by the next round of DNA replication, these lesions will lead to arrested replication and cell death, or to mutations in the descendent cells (either because of incorrect template information or due to lesion bypass by error-prone DNA polymerase) [5].

**Fig 2:**
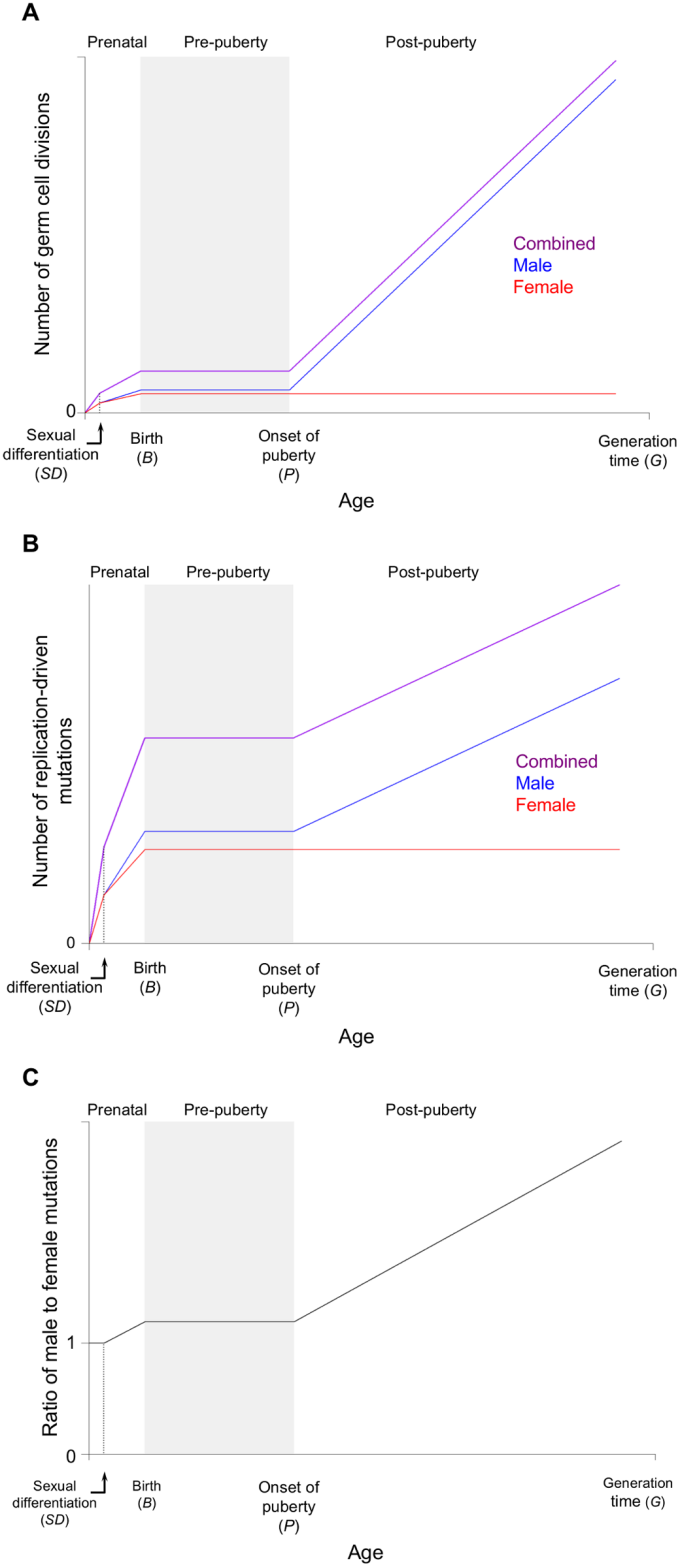
The accumulation of replication-driven mutations with sex and age. (A) An illustration of the increase in the number of germ cell divisions with age in humans. For legibility, the plot is not exactly to scale and the final four cell divisions in males needed to complete spermatogenesis are not shown. The origin is the time of fertilization, and SD, B, P, and G are the times of sexual differentiation, birth, onset of puberty, and reproduction (i.e., generation time), respectively. (B) The increase in the number of mutations due to replication errors with sex and age. (C) The ratio of mutations that occurred in the male versus the female germline (the “male bias”) as a function of increasing parental age.

While the fraction of mutations that is non-replicative in origin remains unknown, the common assumption is that mutations are predominantly replicative [6–9]. The basis for this assumption is a set of observations from disparate fields suggesting that, at least in mammals, mutations seem to track cell divisions. First, in phylogenetic studies, it has been observed repeatedly that species with longer generation times tend to have lower substitution rates, which under neutrality reflects lower mutation rates per unit time (“the generation-time effect”) (e.g., [7,10]). Second, based on comparisons of X, Y chromosomes and autosomes, it has been inferred that substantially more mutations arise in the male than in the female germline (e.g., [6,8,11]). In human genetics, pedigree resequencing studies have confirmed a male bias in mutation of approximately 3:1 at a paternal age of 30, and revealed a linear increase in the number of mutations in the child with the father’s age (e.g., [12,13]). These observations are all qualitatively consistent with mutations arising from the process of copying DNA: all else being equal, organisms with shorter generation times should undergo more germ cell divisions per unit time; in mammals, oocytogenesis is completed by birth whereas spermatogenesis is ongoing since puberty throughout the male lifespan, resulting in more germ cell divisions in males than females (Fig 2A) [14,15].

An informative exception to the “generation time effect” seen in phylogenetic studies is transitions at CpG sites, which represent approximately a fifth of de novo germline mutations [12], and show relatively constant substitution rates across species [16–18]. Their more “clock-like” behavior may reflect their distinct molecular origin [16], as CpG transitions are believed to be due primarily to the spontaneous deamination of the 5-methylcytosine (5mC) [19]. This case demonstrates the potential importance of non-replicative sources in germline mutations and raises the possibility that, despite the usual assumption (e.g., [20,21]), not all non-CpG mutations arise from mistakes in replication.

A third argument for the preponderance of replication errors has been made recently in cancer genetics, on the basis of two observations: (i) that somatic mutations tend to accrue more rapidly in tissues with higher renewal rates [22] and (ii) that, across tissues, the lifetime risk of cancer is associated with the total number of stem cell divisions [9]. Together, these findings were interpreted as indicating that in humans, random errors that occur during DNA replication are the source of most somatic mutations, and hence the main determinant of the odds of developing driver mutations that lead to cancer [9]. However, sequencing of tumor samples also revealed characteristic mutation patterns (“mutational signatures”) that reflect known DNA damage processes by endogenous or exogenous sources [23]. Moreover, environmental mutagens are known to influence the incidence of a subset of cancers, implying a role of mutations of non-replicative origins (e.g., [24,25]). These apparently conflicting observations again point to the importance of understanding how mutations arise in somatic tissues as well as in the germline.

Because, to date, arguments for the replicative origin of mutations have been qualitative and often based on implicit assumptions, we decided to model how the source of mutations relates to their rate of accumulation over cell divisions. For replication-driven mutations, we describe how mutations are expected to accumulate with age, and hence how the generation time relates to the yearly neutral mutation rate. This simple derivation allows us to show that, all else being equal, increases in the generation time will lead to decrease in the mutation rate only under very specific conditions on other parameters. For non-replicative mutations, we relate the mutation rate to rates of DNA damage, repair, and cell division. We show that only when the repair of DNA lesions is highly inefficient will mutations accrue according to absolute time. Otherwise, the accrual of mutations is expected to depend not only on absolute time but also on the rate of cell divisions—a feature previously thought to be specific to replication-driven mutations. By providing explicit expectations for how mutations should accumulate with sex, age, and cell division, these models provide a framework within which to interpret observations from evolutionary biology, human genetics, and cancer genetics.

## Results

### The Accumulation of Mutations Due to Replication Errors

The mutation rate per generation, i.e., the total number of germline mutations between two consecutive generations, is the sum of mutations inherited from both parents, which arose in the lineages of germ cells that gave rise to the child. If mutations are introduced by replication errors, their accumulation will track rounds of DNA replication. In each developmental stage, the number of replication-driven mutations can then be expressed as the product of the number of cell divisions and the mutation rate per cell division. Although a constant mutation rate per cell division is often assumed, explicitly or implicitly [6,26], this need not hold, especially when the cell lineage goes through different development stages, as do germ cells of multicellular organisms. Thus, we consider a more general case, allowing for variation in per cell division mutation rate (e.g., a higher mutation rate in early embryonic development) [27] and describe the accumulation of replication-driven mutations as a piece-wise linear process (following [18]).

For simplicity, we divide germ cell development from fertilization to reproduction into four stages, separated by the settlement of primordial germ cells in the developing gonads (which almost coincides with sexual differentiation), birth, and onset of puberty, respectively. Let *d*_*i*_^*s*^ and *μ*_*i*_^*s*^ be the numbers of cell divisions and replication error rate in the *i*^th^ stage (*i* = 1, 2, 3, 4) in sex *s* (*s* ϵ{*f*,*m*}). Because there is no sex difference in the first stage, *d*_*1*_^*f*^ = *d*_*1*_^*m*^ and *μ*_*1*_^*f*^ = *μ*_*1*_^*m*^, and we replace them by *d*_*1*_ and *μ*_*1*_ (see Table 1 for a list of parameters involved in the model). Previous studies in *Drosophila melanogaster* suggest that the first division of a zygote has an extraordinarily high mutation rate [27,28]. Although the first division in Drosophila is quite distinct from that in mammals, it is possible that it would be more mutagenic in mammals as well, so we consider the first division separately as stage 0, of which the mutation rate is *μ*_*0*_ for both sexes, and re-define stage 1 as from the second post-zygotic division to sex differentiation. The total number of replication-driven autosomal mutations from one parent to the offspring is then:

$$M_{R}^{s}=(\mu_{0}+\mu_{1}d_{1}+\mu_{2}^{s}d_{2}^{s}+\mu_{3}^{s}d_{3}^{s}+\mu_{4}^{s}d_{4}^{s})H,s\epsilon \left\{f,m\right\}$$

where *H* is the total number of base pairs in a haploid set of autosomes.

**Table 1:** A list of parameters used in the model for replication-driven mutations.

Symbol	Definition
*d*_*i*_^*s*^, *μ*_*i*_^*s*^	Number of cell divisions and replication error rate per division in the *i*^th^ stage (*i* = 0, 1, 2, 3, 4) in sex *s* (*s* ϵ{*f*,*m*})
	Stage 0: the first post-zygotic division
	Stage 1: from the second post-zygotic division to sex differentiation
	Stage 2: from sex differentiation to birth
	Stage 3: from birth to puberty
	Stage 4: from puberty to reproduction
*t*_*sg*_,	Duration of spermatogenesis (in years)
*d*_*sg*_	number of cell divisions required to complete spermatogenesis from spermatogonial stem cells
*c* ^*m*^	Number of cell divisions undergone by spermatogonial stem cells in each year
*P*	Age of puberty (assumed to be the same for both sexes)
*G*	Age of reproduction (assumed to be the same for both sexes)
*H*	Total number of base pairs in a haploid set of autosomes
*M*_*R*_^*s*^	Numbers of autosomal replication-driven mutations inherited from the parent of sex *s*
*M*_*R*_	Total number of autosomal replication-driven mutations inherited by an offspring from both parents
*α*_*R*_	Ratio of male to female replication-driven mutations
*m*_*R*,*y*_	Average yearly mutation rate for replication-driven mutations

In mammals, all mitotic divisions of female germ cells are completed by birth of the future mother, so *d*_3_^*f*^ = 0 and *d*_4_^*f*^ = 0, and the total number of replication-driven mutations inherited from mother is (Fig 2B red line):

$$M_{R}^{f}=(\mu_{0}+\mu_{1}d_{1}+\mu_{2}^{f}d_{2}^{f})H.$$ (1)

In contrast, male germ cells undergo divisions in all stages outlined above; furthermore, the number of germ cell divisions after puberty (*d*_*4*_^*m*^) is not a fixed number, because after puberty, sperm are continuously produced through asymmetric division of spermatogonial stem cells, at a roughly constant rate. If we assume that males and females have the same ages of onset of puberty and reproduction (denoted by *P* and *G* respectively), and that a spermatogonial stem cell undergoes *c*^*m*^ divisions each year, the total number of paternal mutations is a function of reproductive age *G* (Fig 2B blue line):

$$M_{R}^{m}=[\mu_{0}+\mu_{1}d_{1}+\mu_{2}^{m}d_{2}^{m}+\mu_{3}^{m}d_{3}^{m}+\mu_{4}^{m}(c^{m}(G-P-t_{sg})+d_{sg})]H,$$ (2)

where *t*_*sg*_ and *d*_*sg*_ are the time (in years) and the number of cell divisions needed to complete spermatogenesis from spermatogonial stem cells. The two divisions in meiosis are counted as one here, because only one round of DNA replication takes place in meiosis.

Summing Eqs 1 and 2, the total number of autosomal replication-driven mutations inherited by a diploid offspring from both parents is (Fig 2B purple line):

$$M_{R}=M_{R}^{f}+M_{R}^{m}=[2\mu_{0}+2\mu_{1}d_{1}+\mu_{2}^{f}d_{2}^{f}+\mu_{2}^{m}d_{2}^{m}+\mu_{3}^{m}d_{3}^{m}+\mu_{4}^{m}(c^{m}(G-P-t_{sg})+d_{sg})]H.$$

By dividing Eq 2 by Eq 1, we obtain the ratio of male to female replication-driven mutations:

$$\alpha_{R}=\frac{M^{m}}{M^{f}}=\frac{\mu_{0}+\mu_{1}d_{1}+\mu_{2}^{m}d_{2}^{m}+\mu_{3}^{m}d_{3}^{m}+\mu_{4}^{m}d_{sg}}{\mu_{0}+\mu_{1}d_{1}+\mu_{2}^{f}d_{2}^{f}}+\frac{\mu_{4}^{m}c^{m}}{\mu_{0}+\mu_{1}d_{1}+\mu_{2}^{f}d_{2}^{f}}\cdot (G-P-t_{sg}),$$

which suggests that, keeping other parameters unchanged, increases in generation time *G* will lead to a stronger male bias in mutation, as expected intuitively (Fig 2C).

It follows that the average yearly mutation rate (i.e., the substitution rate if all mutations are neutral) is a function of *G*:

$$m_{R,y}=\frac{m_{R,g}}{G}=\frac{2\mu_{0}+2\mu_{1}d_{1}+\mu_{2}^{f}d_{2}^{f}+\mu_{2}^{m}d_{2}^{m}+\mu_{3}^{m}d_{3}^{m}+\mu_{4}^{m}d_{sg}+\mu_{4}^{m}c^{m}(G-P-t_{sg})}{2G}\cdot$$ (3)

In order to explore the effect of generation time on the average yearly mutation rate, it is useful to reorganize Eq 3 as:

$$m_{R,y}=\frac{\mu_{4}^{m}c^{m}}{2}+\frac{A*}{2G},$$ (4)

where $A^{*}=2\mu_{0}+2\mu_{1}d_{1}+\mu_{2}^{f}d_{2}^{f}+\mu_{2}^{m}d_{2}^{m}+\mu_{3}^{m}d_{3}^{m}-\mu_{4}^{m}(c^{m}P+c^{m}t_{sg}-d_{sg}),$ which is independent of *G*.

Eq 4 suggests that if and only if A* = 0 will the yearly mutation rate be independent of *G*. Otherwise, *m*_R,y_ will either increase or decrease monotonically with *G*, depending on the sign of A*. Changes in the timing of puberty (*P*), in the number of cell divisions (*d*_*i*_^*s*^) and in the replication error rate per cell division in each stage (*μ*_*i*_^*s*^) will also influence the dependence of *m*_R,y_ on *G*.

**Fig 3:**
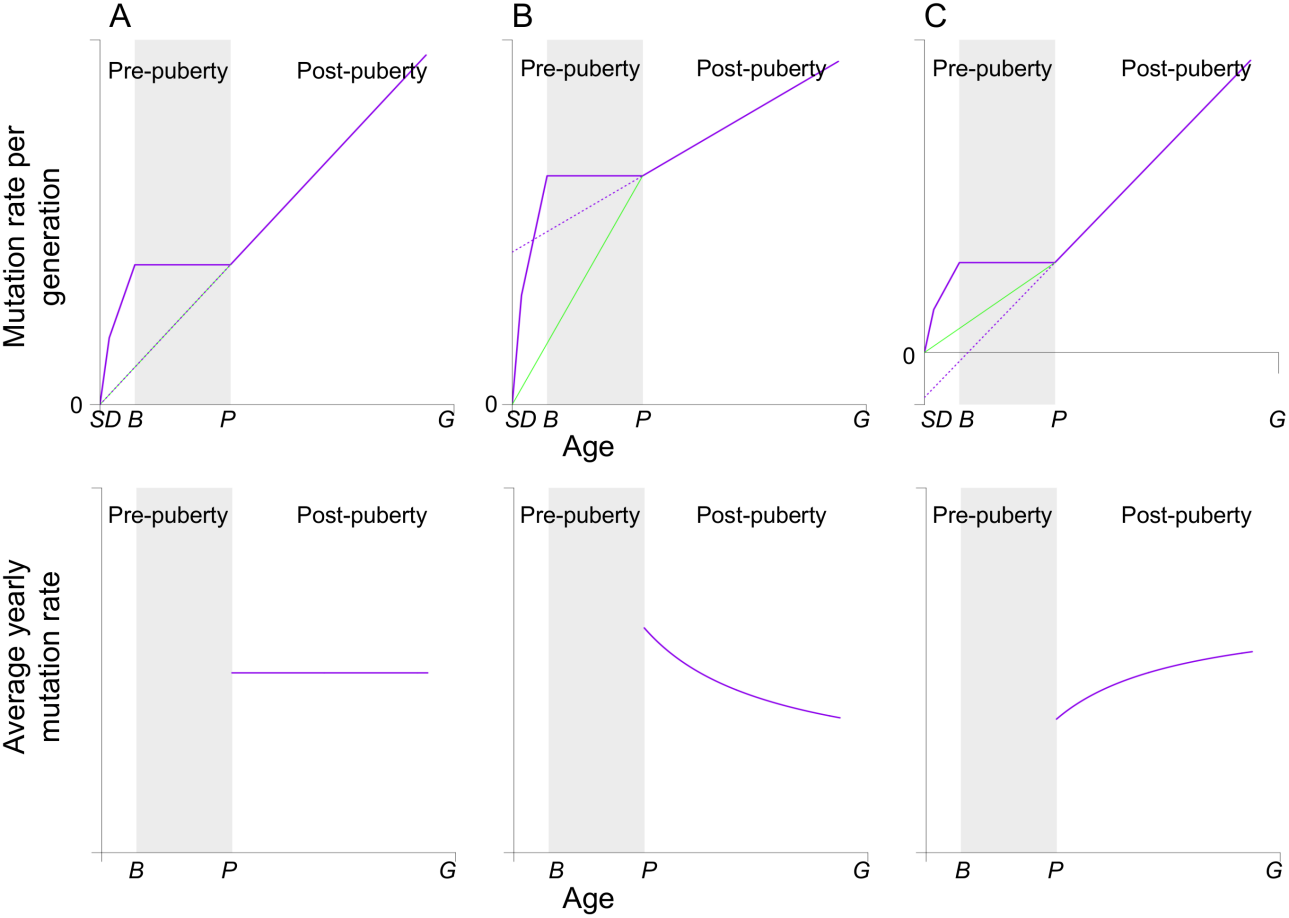
The effect of the generation time on the sex-averaged yearly rate of replication-driven mutations. The sex-averaged mutation rate per generation (solid purple line) increases with the generation time (assumed to be the same for males and females). Depending on the age of puberty (*P*), generation time (*G*), and the per cell division mutation rates, a linear fit to the number of mutations after puberty (dotted purple line) could have a zero, positive, or negative intercept at age zero, and the slope of this linear fit represents the yearly mutation rate after puberty. The slope of the green line represents the average yearly mutation rate prior to puberty. The effect of *G* on the overall average yearly mutation rate (*m*_R,y_) depends on the relative values of the two slopes, which is equivalent to the sign of the intercept of dotted purple line at age zero: (A) If the intercept is zero, the dotted purple and green lines coincide, and the yearly mutation rates before and after puberty are equal, so the *G* does not affect *m*_R,y_. (B) If the intercept is positive, the yearly mutation rate after puberty is smaller than that before puberty, so *m*_R,y_ decreases with generation time. (C) If the intercept is negative, the yearly mutation rate after puberty is greater than that before puberty, so *m*_R,y_ increases with generation time.

The relationship between *m*_R,y_ and *G* can also be directly read off the curve in Fig 3. The mutation rate per generation increases linearly with *G* after puberty, but this linear relationship does not apply to the period before puberty. If and only if the extended fitted line passes through the origin will the mutation rate per generation be exactly proportional to the generation time, and the average yearly mutation rate unaffected by *G*. If the intercept of the extrapolated line at age zero is positive, *m*_R,y_ *decreases* with *G*, consistent with the observed “generation time effect” in primates. Conversely, if the intercept is negative, *m*_R,y_ *increases* with *G*. In fact, the intercept obtained by extrapolation is exactly A* in Eq 3, so interpretation from Fig 3 is equivalent to that suggested by Eq 4.

Although estimates of other parameters exist, little is known about the replication error rate per cell division in germ cells, so it is unclear whether A* is positive or negative. However, it seems highly coincidental that an expression that involves multiple variables would happen to equal zero. Therefore, we argue that there is almost certainly an effect of generation time on yearly mutation rate in humans, although the magnitude of the effect could be small. The magnitude of the paternal age effect in pedigree data suggests that there should be generation-time effect in humans (see S1 Text).

Our model further reveals that, all else being equal, a longer generation time can lead to either an increase or decrease in the average yearly rate at which replicative mutations accrue. Therefore, the general observation that substitution rate in mammals tends to *decrease* with increasing generation times [7,10,16] is not necessarily expected; in fact, its existence requires very specific conditions on ontogenesis to hold (shown in Fig 3B). Moreover, given the current understanding of germ cell development in humans, the generation-time effect implies a higher mutation rate per cell division in early embryonic development than in spermatogenesis (see S1 Text for a discussion of available data in humans and chimpanzees).

Since mammalian species differ drastically in life history traits as well as development and renewal processes of germ cells [26,29], Eq 4 implies that the yearly mutation rate likely varies among species (even if per cell division mutation rates remain constant). As a result, unless life history traits co-vary in certain ways, we should not expect neutral substitution rates to be constant across mammalian species—or even along single evolutionary lineages. An important implication is that changes in life history among hominins [30] introduce uncertainty about dates in human evolution obtained under the assumption of a molecular clock [31].

### The Accumulation of Non-replicative Mutations with Cell Divisions

DNA is subject to large numbers of damaging events every day as a result of normal cellular metabolism, and more DNA lesions may be generated by exogenous agents [32]. Typical DNA damage includes depurination and deamination due to DNA hydrolysis; alkylation and oxidation of bases induced by chemicals such as ethylmethane sulfonate or reactive oxygen species; pyrimidine dimers caused by ultraviolet radiation; and single- or double-stranded breaks produced by gamma and X-rays. Most single-stranded lesions cannot pair properly with any regular bases (termed “noncoding lesions”) and thus will block DNA replication if unrepaired (Fig 1). However, a few alterations to nucleotides can pair with bases different from the original Watson-Crick partners; such lesions (termed “miscoding lesions”), if unrepaired before replication, will lead to irreversible replacement of a base pair after cell division (Fig 1) [5].

To model the accrual of non-replicative mutations, we start by considering deamination of methylated CpG sites, which is the best understood example of miscoding lesions, and discuss more complex mutagenesis mechanisms in the S2 Text. This modification turns the methylated cytosine (mC) into a thymine (T); if uncorrected before DNA replication, an adenine instead of a guanine will be incorporated into the nascent strand, which results in a mutation in one of the two daughter cells. While DNA replication and cell division are obviously two distinct events, they are tightly coordinated such that DNA is replicated exactly once before each division (other than in meiosis and under a few unusual conditions). In what follows, we therefore do not distinguish between the two events.

**Fig 4:**
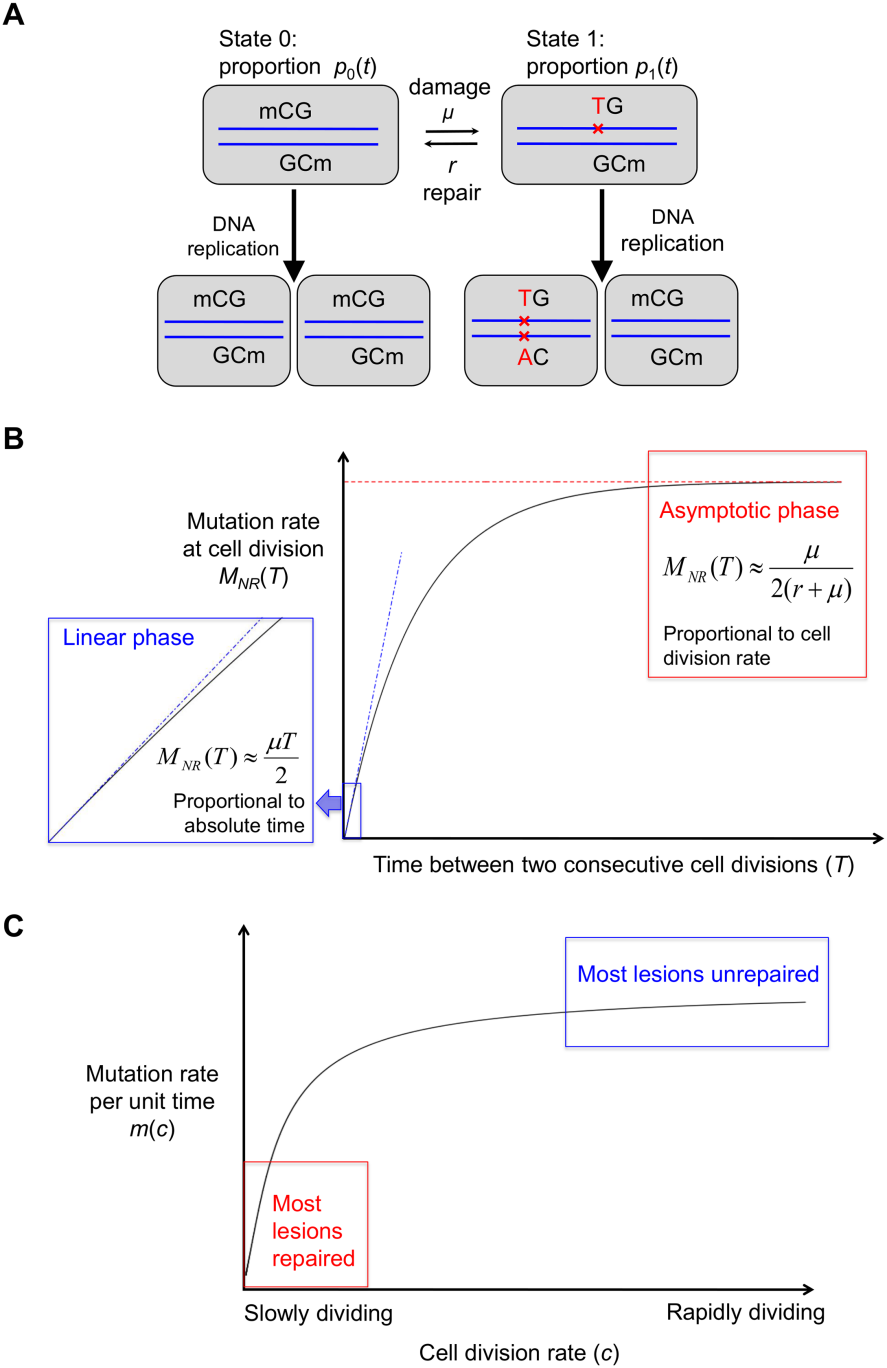
The basic model for non-replicative mutations. (A) The DNA dynamics before and after cell division. The upper panel shows the DNA states prior to the next cell division, and the lower panel shows the DNA states of the daughter cells after cell division. (B) The per cell division mutation rate increases with the time between two consecutive cell divisions and reaches an asymptote when the cell divides sufficiently slowly. (C) The rate at which non-replicative mutations accumulate per unit time increases with the cell division rate.

We model the proportion of damaged base pairs at the time of cell division by considering the effects of both damage and repair (Fig 4A). For simplicity, we assume that single-strand damage occurs at a constant instantaneous rate *μ* throughout cell cycle and that the repair machinery recognizes lesions at a constant rate *r* (Fig 4A). Thus, the proportion of base pairs that carry a lesion at time *t* after the last cell division, *p*_1_(*t*), is described by a simple differential equation:

$$\frac{dp_{1}}{dt}=\mu (1-p_{1})-rp_{1},$$

with the initial condition *p*_1_(0) = 0.

The solution to the differential equation is:

$$p_{1}(t)=\frac{\mu }{\mu +r}(1-e^{-(\mu +r)t}).$$

Because each unrepaired single-strand lesion leads to a base pair substitution in one of the two daughter cells, the average mutation rate in one cell division (i.e., the expected fraction of base pairs that differ between a daughter cell and its mother cell) is:

$$M_{NR}(T)=\frac{1}{2}p_{1}(T)=\frac{\mu }{2(\mu +r)}(1-e^{-(\mu +r)T}),$$ (5)

where *T* is the time between two consecutive cell divisions (Fig 4B).

We assume that *μ*<<1/*T* for any biologically reasonable value of *T*, so even in the absence of DNA repair, the absolute mutation rate per base pair per cell division (≈½*μT*) is very small. In addition, we focus on a single cell lineage and assume an infinite sites model, in which each genomic site can be mutated at most once. Thus, the total mutation rate over many cell divisions is simply the sum of the mutation rates for every division.

A key feature of the result in Eq 5 is that the accumulation of mutations per cell division exhibits two different limiting behaviors, depending on the relative rates of cell division and repair. When the rate at which lesions are repaired is much slower than the rate of cell division (*rT*<<1), the number of mutations is approximately proportional to time between two rounds of DNA replication:

$$M_{NR}(T)=\frac{\mu T}{2}.$$ (6)

The intuition is that, for a cell under this condition, there is almost no time for the repair machinery to correct lesions, so almost all lesions result in mutations. Consequently, mutations accumulate at a constant rate regardless of the rates of cell division and repair (Fig 4B, red box). In other words, non-replicative mutations that are inefficiently repaired will track absolute time.

In contrast, in the other limit where the repair is highly efficient relative to the rate of cell division (*rT*>>1), the number of mutations approaches an equilibrium level by the time of cell division:

$$M_{NR}(T)=\frac{\mu }{2(\mu +r)}.$$ (7)

As a result, mutations accumulate at a rate that is roughly proportional to the number of cell divisions, regardless of absolute time (Fig 4B, blue box). Here, the intuition is that when repair is highly efficient, the few lesions that have not been corrected tend to be those that arose right before the cell division, and therefore the time since the last division has little effect. Importantly, under this scenario, the accrual of mutations that arise from lesions mimics what would be expected from replication errors. We note that the existence of such an equilibrium comes from the assumption of no error in repair; however, even when errors in repair are taken into consideration, there exists a phase in which repair and damage roughly balance out, so the mutation rate is proportional to the cell division rate (see S2 Text).

To understand how the mutation rate of non-replicative mutations depends on absolute time and the rate of cell division in general, we derive the mutation rate per unit time as the product of mutation rate per cell division and the cell division rate (*c* = 1/*T*>0):

$$m(c)=cM_{NR}(\frac{1}{c})=\frac{c}{2(1+R)}(1-e^{-\frac{(1+R)\mu }{c}}).$$ (8)

The mutation rate *m*(*c*) has two limiting behaviors when *c* approaches infinity and zero, respectively, which have the same intuitive explanations as Eqs 6 and 7, respectively. Moreover, it can be shown that *m*(*c*) is a concave increasing function of *c*. In other words, in a given period of time, faster dividing cell lineages accumulate more non-replicative mutations than slowly dividing lineages, but the increase in the number of mutations is smaller than the increase in the cell division rate. Therefore, when repair is neither inefficient nor extremely efficient, and given fixed damage and repair rates, faster dividing lineages are expected to accumulate non-replicative mutations at a higher rate per year than more slowly dividing ones (Fig 4C and see Table 2 for a list of parameters involved in the model).

**Table 2:** A list of parameters used in the model for non-replicative mutations.

Symbol	Definition
*μ*	Instantaneous damage rate
*r*	Instantaneous repair rate
*R = r/μ*	Relative repair rate compared to damage rate
*p*_*0*_(*t*)	Proportion of base pairs in the genome that do not carry a lesion at time *t* since last cell division
*p*_*1*_(*t*)	Proportion of base pairs in the genome that carry a single-strand lesion at time *t* since last cell division
*T*	Time between two consecutive divisions of a cell lineage
*M*_*NR*_(*T*) = ½ *p*_*1*_(*T*)	Mutation rate per division for a cell that divides every *T* unit of time
*c* = 1/*T*	Cell division rate
*m*(*c*) = c*M_*NR*_ (*1/c*)	Mutation rate per unit time for a cell with cell division rate *c*

This model can be extended readily to incorporate more features, such as other types of non-replicative mutations as well as to understand phenomena such as the strand bias in mutations associated with transcription (see S2 Text) [33,34]. Although the quantitative results differ, the main conclusion holds: the accumulation of non-replicative mutations depends critically on the repair efficiency in relation to the cell division rate.

## Discussion

**Fig 5:**
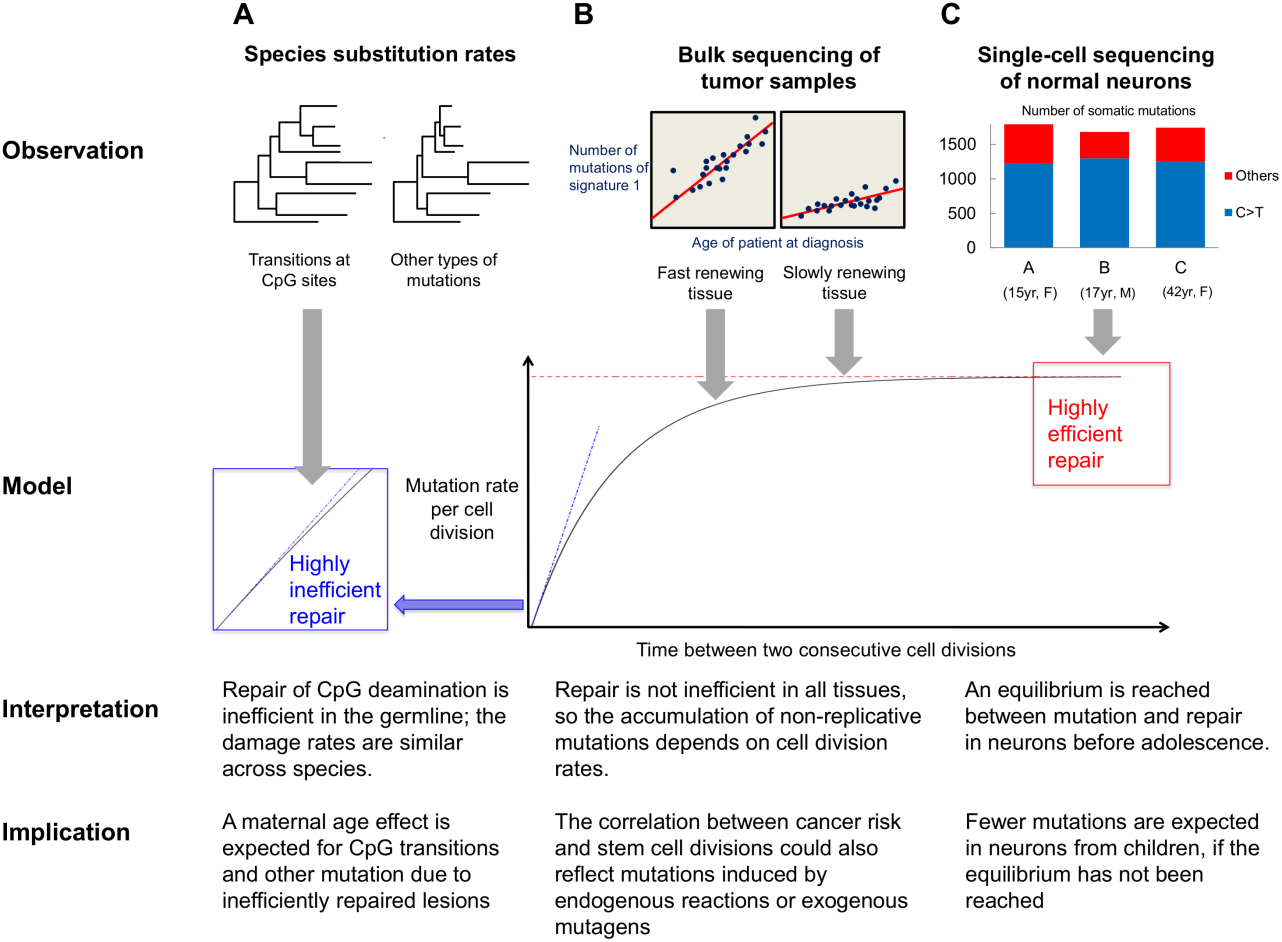
A visual summary of interpretation of existing observations based on our model and further predictions about germline and somatic mutations in humans.

These results demonstrate the fundamental importance of repair efficiency in determining the dependence of mutation rates on age, sex, and cell division rate (Fig 5). When DNA repair is inefficient, we should expect a linear accumulation of damage-induced mutations, partially justifying the expectation that neutral substitution rates of non-replicative mutations should not depend on generation time or other life history traits, and hence may be constant across species. However, our model highlights additional conditions for this expectation to be met: in particular, it reveals that the clock-like behavior of CpG transitions in mammals not only requires a non-replicative origin but also implies both relatively low repair efficiency in germ cells and similar damage rates across mammalian species (Fig 5A).

A further implication is that the number of mutations of maternal origin should increase with the mother’s age for CpG transitions and other mutations that arise from inefficiently repaired lesions. In this regard, we speculate that the current lack of a detectable maternal age effect may be due to underpowered sample sizes (notably because of the strong correlation between maternal and paternal ages). In any case, our model predicts that a maternal age effect should be detectable with sufficient data and reliable identification of parental origin of mutations (e.g., by sequencing of a third generation). Conversely, the detection of a maternal age effect on mutation rate would provide prima facie evidence for the existence of non-replicative mutations that are not efficiently repaired (assuming no relationship between the age at which an oocyte is ovulated and the number of cell divisions experienced during oocytogenesis [35]).

Also of note, lesions that have the same damage rate but are recognized by distinct repair mechanisms may differ not only in their absolute mutation rates but also in their time dependencies. Indeed, changes to the repair efficiency (or to the division rate) could alter the sex and time dependence of non-replicative mutations; for example, decreases in repair efficiency could lead mutations that previously tracked cell division rates to depend more on absolute time. Therefore, the phylogenetic molecular clock should not necessarily run at a steady rate even for mutations due to spontaneous DNA damage.

Our modeling results also shed light on studies of somatic mutations. As an illustration, a recent single-cell sequencing study identified mutations in neurons from the cerebral cortex of three healthy individuals [36]. The numbers of mutations in each cell were similar regardless of the donor’s sex and age (ranging from 15 to 42 years, Fig 5C) [37]. The genome-wide distribution of the somatic mutations appeared to be associated with transcription, with most identified mutations being C to T transitions at methylated cytosines. These observations led the authors to conclude that the mutations that they observed were due to non-replicative damage that was poorly repaired [36]. However, if mutations are non-replicative in origin and not repaired, more DNA lesions should accrue in older individuals, even in post-mitotic cells. In light of our model, an explanation is that an equilibrium between DNA damage and repair was reached before adolescence, and thus that the number of mutations does not increase further with age (Fig 5C). If this is the case, then there should be fewer somatic mutations in post-mitotic neurons from younger individuals, in which the equilibrium has not been reached.

Similarly, the model helps to interpret patterns observed in tumor samples, in which the total number of somatic mutations increases with the age of patient at diagnosis and grows at higher rates in fast renewing tissues [22]. Deamination at CpG sites make substantial contribution to mutations in almost all cancer types and accumulate at constant yearly rates that appear to be positively correlated with the turnover rates of the corresponding normal tissues (Fig 5B) [23,38]. As we have shown, all else being equal, a positive correlation is expected even for mutations that arise from DNA damage, so long as lesions are not poorly repaired in all somatic tissues.

Importantly, then, the recently reported correlation between number of stem cell divisions and lifetime risk of cancer across tissues is consistent with mutations of both replicative and non-replicative origins, and does not provide any evidence that most mutations are attributable to replication mistakes in stem cell divisions (what the authors referred to as “bad luck” in [9]). Of course, tumorigenesis is a multistep process that depends not only on the accumulation of mutations but also on tissue architecture as well as the order and consequences of specific mutational events, and gaining insight into its causes will likely require consideration of all these facets. What our model makes apparent is that it will also be important to incorporate a realistic model for the source of mutations.

Similar arguments apply to the male bias in mutation found by resequencing pedigrees and the generation time effect in phylogenetics: neither observation provides evidence for a replication-driven mutational process, as they could also reflect mutations arising from residual lesions left after efficient repair. Given these considerations, it becomes clear that, based on available data, we still do not know if a substantial proportion of human germline and somatic mutations—including those at non-CpG sites—are non-replicative in origin.

In summary, we introduce a model that helps to interpret findings from studies of somatic mutations, human pedigrees, and phylogenies. Although very simple, its behavior appears to be robust. By making explicit the relationship between the genesis of mutations and their accumulation over ontogeny, the model reveals the critical importance of both the source of mutations and the repair efficiency of lesions. Because replicative mutations and non-replicative mutations can display similar properties when repair is efficient, none of the previous observations of correlations between mutation and cell division rates lends strong support to the commonly held belief that most mutations are replicative in origin. Further experimental work is therefore needed to distinguish between different sources of mutation. Notably, fitting models such as this one to growing data from diverse fields should provide a quantitative understanding of how DNA changes accumulate in somatic tissues during a lifetime and in the germline over evolutionary time scales.

## Supporting Information

S1 Fig A model for non-replicative mutations with errors in repair. (A) The DNA dynamics with errors in repair can be described by three states. The upper panel shows the DNA states prior to the next cell division, and the lower panel shows the DNA states of the daughter cells after cell division. (B) The proportion of base pairs without lesion (*p*_0_(*t*)), the proportion of base pairs with single-strand lesions (*p*_1_(*t*)) and the mutation rate per cell division (*M*_*NR*_(*t*)) as functions of the time since the last division. Same values of the damage and repair rates are used for all cases with repair. In the case with no DNA repair, the value of *r* is set to zero. (C) Log-log plots for *p*_0_(*t*), *p*_1_(*t*), and *M*_*NR*_(*t*). The dotted blue lines show the boundaries between the four phases for the case with *ɛ* = 0.0001 (represented by the blue curve). Notice that both axes are on a logarithmic scale, so later phases should be longer than they appear on the plot.
(PDF)

S1 Table A list of parameters and estimated values used in the model for replication-driven mutations. See S1 Text for references behind each parameter value.
(DOC)

S2 Table A list of parameters used in the model of more complex scenarios of non-replicative mutations. (DOC)

S1 Text The predicted generation time effects in humans and chimpanzees, based on available data. (DOC)

S2 Text More complex scenarios of mutations that arise from DNA lesions. (DOC)
