# Supplementary figures and images for: Interpreting the Dependence of Mutation Rates on Age and Time
Source: PLoS Biol. 2016 Jan 13;14(1):e1002355. doi: 10.1371/journal.pbio.1002355 (PMC4711947; doi:10.1371/journal.pbio.1002355)

**A**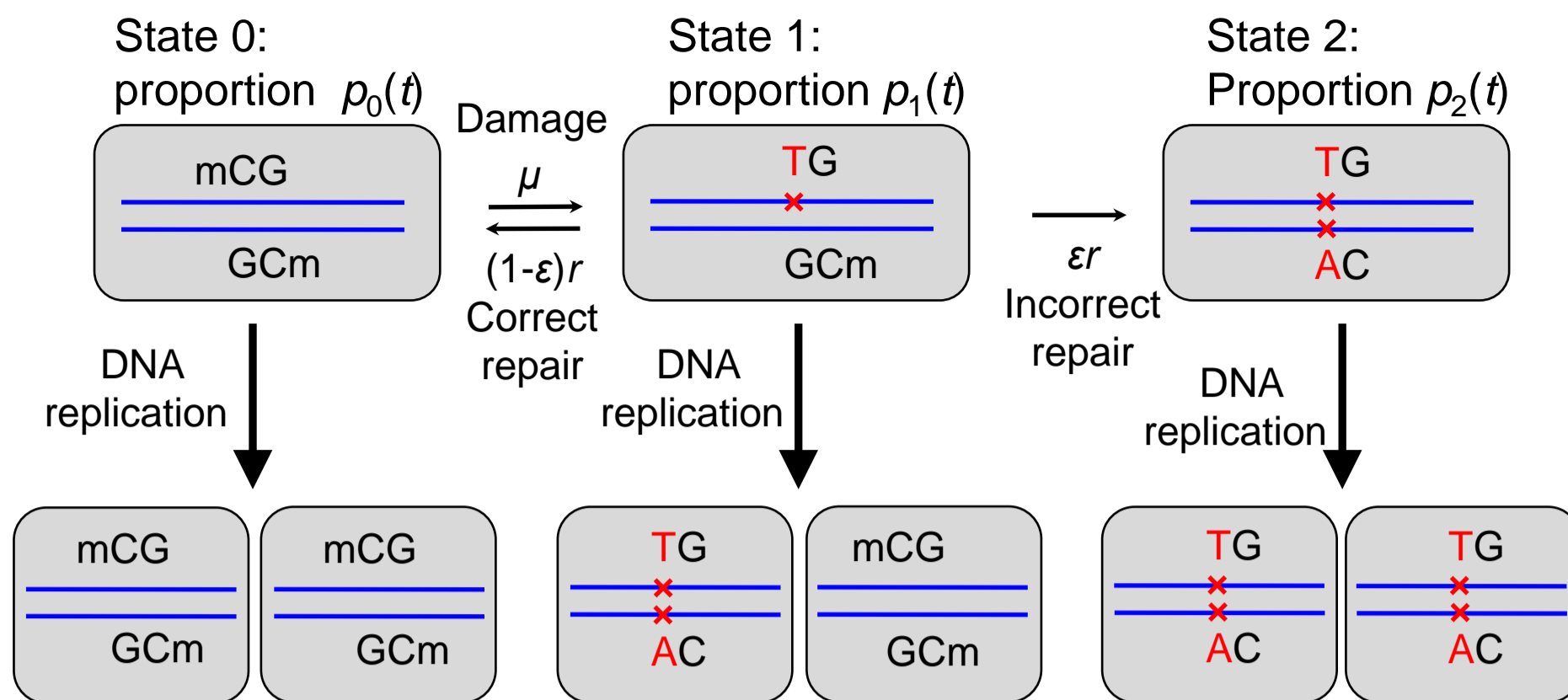**B**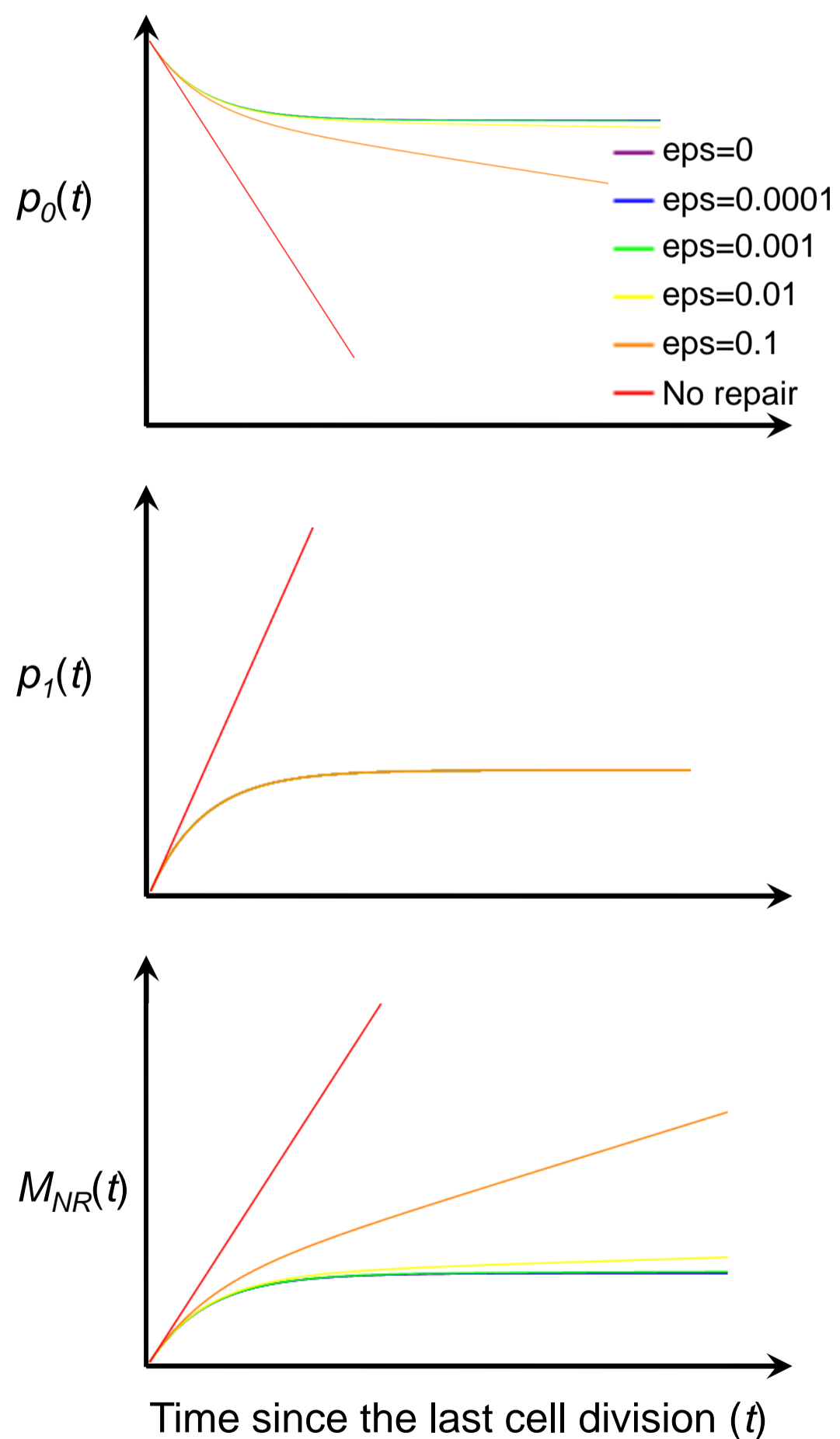**C**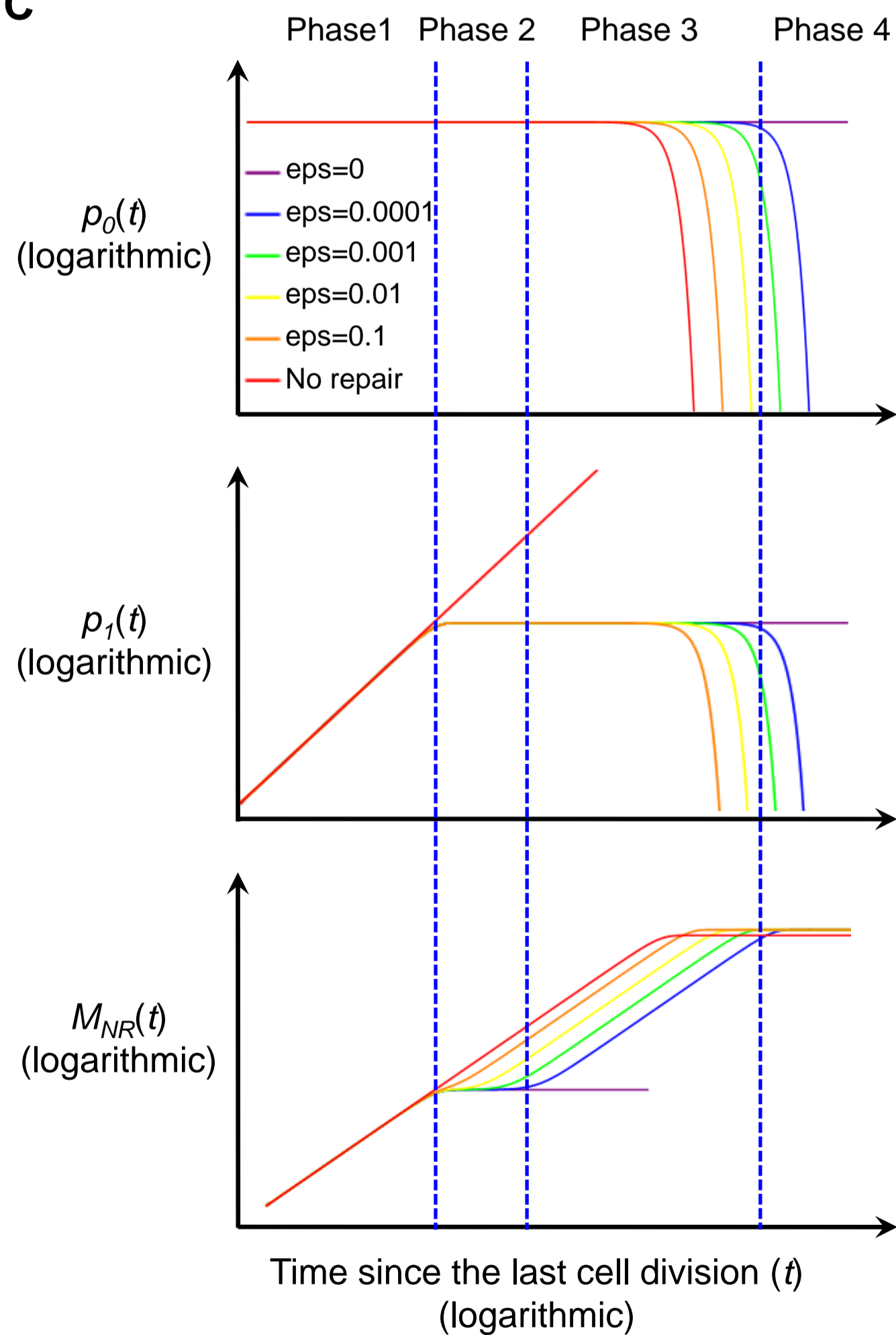

Supplement: S1 Fig — (A) The DNA dynamics with errors in repair can be described by three states. The upper panel shows the DNA states prior to the next cell division, and the lower panel shows the DNA states of the daughter cells after cell division. (B) The proportion of base pairs without lesion (p0(t)), the proportion of base pairs with single-strand lesions (p1(t)) and the mutation rate per cell division (MNR(t)) as functions of the time since the last division. Same values of the damage and repair rates are used for all cases with repair. In the case with no DNA repair, the value of r is set to zero. (C) Log-log plots for p0(t), p1(t), and MNR(t). The dotted blue lines show the boundaries between the four phases for the case with ɛ = 0.0001 (represented by the blue curve). Notice that both axes are on a logarithmic scale, so later phases should be longer than they appear on the plot. (PDF) [file pbio.1002355.s001.pdf]
