# Supplementary material for: Interpreting the Dependence of Mutation Rates on Age and Time
Source: PLoS Biol. 2016 Jan 13;14(1):e1002355. doi: 10.1371/journal.pbio.1002355 (PMC4711947; doi:10.1371/journal.pbio.1002355)
Supplement: S1 Table — See S1 Text for references behind each parameter value. (DOC) [file pbio.1002355.s002.doc]

| **Symbol** | **Definition** | **Estimate** |
| --- | --- | --- |
| *d_i_^s^*, *μ_i_^s^* | Number of cell divisions and replication error rate per division in the *i*^th^ stage (*i* =0, 1, 2, 3, 4) in sex *s* ($s\in\{f,m\}$).  Stage 0: the first post-zygotic division;  Stage 1: from the second post-zygotic division to sex differentiation;  Stage 2: from sex differentiation to birth;  Stage 3: from birth to puberty;  Stage 4: from puberty to reproduction. | *d_1_* = 15;  *d_2_^m^* = 21;  *d_2_^f^* = 15;  *d_3_^m^* = 0;  *d_3_^f^* = 0;  *d_4_^f^* = 0;  *d_4_^m^* = *c ^m^*(*G*-*P- t_sg_*)+ *d_sg_* |
| *t_sg_,* | Duration of spermatogenesis (in years) | *t_sg_* = 0.2 |
| *d_sg_* | number of cell divisions required to complete spermatogenesis from spermatogonial stem cells | *d_sg_* = 4 |
| *c ^m^* | Number of cell divisions undergone by spermatogonial stem cells in each year | *c ^m^* = 23 |
| *P* | Age of puberty (assumed to be the same for both sexes) | *P* = 13 |
| *G* | Age of reproduction (assumed to be the same for both sexes) |  |
| *H* | Total number of base pairs in a haploid set of autosomes |  |
| *M_R_^s^* | Numbers of autosomal replication-driven mutations inherited from the parent of sex *s* |  |
| *M_R_* | Total number of autosomal replication-driven mutations inherited by an offspring from both parents |  |
| *m_R,g_* | Per generation mutation rate for replication-driven mutations |  |
| *α_R_* | Ratio of male to female replication-driven mutations |  |
| *m_R,y_* | Average yearly mutation rate for replication-driven mutations |  |
