# Supplementary material for: Interpreting the Dependence of Mutation Rates on Age and Time
Source: PLoS Biol. 2016 Jan 13;14(1):e1002355. doi: 10.1371/journal.pbio.1002355 (PMC4711947; doi:10.1371/journal.pbio.1002355)
Supplement: S2 Table — (DOC) [file pbio.1002355.s003.doc]

| Symbol | Definition |
| --- | --- |
| *μ* | Instantaneous damage rate |
| *r* | Instantaneous repair rate |
| *R=μ/r* | Relative repair rate compared to damage rate |
| *p_0_*(*t*) | Proportion of base pairs in the genome that do not carry a lesion at time *t* since last cell division |
| *p_1_*(*t*) | Proportion of base pairs in the genome that carry a single-strand lesion at time *t* since last cell division |
| *p_2_*(*t*) | Proportion of base pairs in the genome that substituted at time *t* since last cell division |
| *T* | Time between two consecutive divisions of a cell lineage |
| *M_NR_*(*T*) = ½ *p*_1_(*t*)+ *p*_2_(*t*) | Mutation rate per division for a cell that divides every *T* unit of time |
| *c*=1/*T* | Cell division rate |
| *m*(*c*) = *c***M_NR_* (*1/c*) | Mutation rate per unit time for a cell with cell division rate *c* |
| *ɛ* | Error rate of repair |
