# Supplementary material for: Interpreting the Dependence of Mutation Rates on Age and Time
Source: PLoS Biol. 2016 Jan 13;14(1):e1002355. doi: 10.1371/journal.pbio.1002355 (PMC4711947; doi:10.1371/journal.pbio.1002355)
Supplement: S1 Text — (DOC) [file pbio.1002355.s004.doc]

***1. Properties of germline mutations inferred from human trio studies suggest the yearly mutation rate should decrease with generation time***

One way to test whether there exists a generation time effect in humans is to extend the fitted line of empirical *de novo* mutation data in order to estimate the intercept at age zero. When we combine data from three available whole-genome datasets [1-3] in order to consider the effect of paternal age on mutation rates, the intercept of the linear regression line is significantly positive [4]. This suggests that a generation time effect might be operating in humans (at least in the populations under study).

The positive intercept also has interesting implications for the per cell division mutation rates in different development stages of germ cells in humans. As shown in equation (4) in the main text, there is a positive intercept if and only if:

$2\mu_{0}+2\mu_{1}d_{1}+\mu_{2}^{f}d_{2}^{f}+\mu_{2}^{m}d_{2}^{m}+\mu_{3}^{m}d_{3}^{m}>\mu_{4}^{m}(c^{m}P+c^{m}t_{sg}-d_{sg})$.

It is estimated that, in humans, *d_1_*=15, *d_2_^f^*=15, *d_2_^m^*=21, *d_3_^m^*=0, *P*=13, *t_sg_*=0.2, *d_sg_*=4 and *c^m^*=23 [5, 6; see Table 1 for a list of parameters and their estimated values]. By plugging in these estimates, we obtain:

$2\mu_{0}+30\mu_{1}+15\mu_{2}^{f}+21\mu_{2}^{m}>(23\times13.2-4)\mu_{4}^{m}=303.6\mu_{4}^{m}$. (9)

(Equations are numbered following the main text.)

Because the coefficient in front of *μ_4_^m^* is so large, inequality (9) cannot hold if$\mu_{0}$,$\mu_{1}$, $\mu_{2}^{f}$and$\mu_{2}^{m}$are all smaller than or equal to$\mu_{4}^{m}$. In other words, at least one of the four pre-puberty per cell division mutation rates has to be much er than the post-puberty rate in males.

One possibility is that the first division in the zygote has an extremely high mutation rate, while all other cell divisions share similar mutation rates, as appears to be the case in a completely different developmental context, in *Drosophila melanogaster* [7, 8]. If we assume that$\mu_{1}=\mu_{2}^{f}=\mu_{2}^{m}=\mu_{4}^{m}$, then inequality (9) reduces to:

$\mu_{0}>117\mu_{1}$,

which is surprisingly high, but on par with what is seen in *D. melanogaster*, where the first post-zygotic division is estimated to be ~800 fold more mutagenic than other development stages of the embryogenesis [8].

In addition to an extraordinarily high mutation rate for the first cell division, other scenarios (for example,$\mu_{0}=\mu_{1}=\mu_{2}^{f}=\mu_{2}^{m}>4.46\mu_{4}^{m}$) could also explain a positive intercept at age zero. More data from humans is needed to distinguish between these scenarios. Also needed are better estimates of numbers of germ cell divisions in each development stage in humans, as current knowledge about human spermatogenesis largely comes from one study in the early 1960s, which determined SSC division cycle by radioautograph of testicular biopsies after injection of tritiated thymidine [9].

***2. Properties of germline mutations inferred from a chimpanzee pedigree suggest that the yearly mutation may increase with generation time***

A recent pedigree study in Western chimpanzees provides the opportunity to evaluate the presence and magnitude of a generation effect on *de novo* mutation rate in a non-human great ape species. Intriguingly, the intercept of mutation accumulation curve at age zero appears to be negative, although not significantly different from zero. If this finding were to hold up with more data, it would imply that the substitution rate should stay relatively constant or even *increase* with generation time, i.e., that chimpanzees would show the opposite of the generation time effect. It is thought that chimpanzees reach puberty at an earlier age (*P*=8.75 since conception) [10] and have shorter duration of spermatogenesis and spermatogenic cycles compared to humans (*t_sg_*=0.17, ) [11], but data on the number of cell divisions in each development stage are much more scarce. If we assume that the development process is conserved between humans and chimpanzees until birth and use the estimated numbers of cell divisions in humans as proxies for those of chimpanzees, the non-significant negative intercept at age zero indicates that:

$2\mu_{0}+30\mu_{1}+15\mu_{2}^{f}+21\mu_{2}^{m}\leq(26\times8.58-4)\mu_{4}^{m}=219\mu_{4}^{m}$. (10)

Taking these point estimates at face value, the comparison between the results of studies in the two species leads to two intriguing findings. First, comparing (9) and (10) suggests that the mutation rates per cell division cannot all be the same for the two species under the assumption of conserved germ cell development process, indicating that either the mutation rates or the numbers of cell divisions during various stages have evolved between these two closely related species. Second, despite distinct onsets of puberty and the different relationships between the average generation time and the number of *de novo* mutations, the average yearly mutation rates are remarkably similar for humans and Western chimpanzees. However, the sample size for the chimpanzee study is very small, and the captive animals’ reproductive ages differ substantially from wild animals, so further data are required to assess if these conclusions are solid. What is underscored by this comparison, however, is that a decrease in the mutation rate with increasing generation time (i.e., the “generation time effect”) is by no means a given, and in fact requires quite specific conditions to hold.

**References**

1. Campbell CD, Chong JX, Malig M, Ko A, Dumont BL, Han L, et al. Estimating the human mutation rate using autozygosity in a founder population. Nature genetics. 2012;44(11):1277-81. Epub 2012/09/25. doi: 10.1038/ng.2418. PubMed PMID: 23001126; PubMed Central PMCID: PMC3483378.

2. Michaelson JJ, Shi Y, Gujral M, Zheng H, Malhotra D, Jin X, et al. Whole-genome sequencing in autism identifies hot spots for de novo germline mutation. Cell. 2012;151(7):1431-42. Epub 2012/12/25. doi: 10.1016/j.cell.2012.11.019. PubMed PMID: 23260136; PubMed Central PMCID: PMC3712641.

3. Jiang YH, Yuen RK, Jin X, Wang M, Chen N, Wu X, et al. Detection of clinically relevant genetic variants in autism spectrum disorder by whole-genome sequencing. American journal of human genetics. 2013;93(2):249-63. Epub 2013/07/16. doi: 10.1016/j.ajhg.2013.06.012. PubMed PMID: 23849776; PubMed Central PMCID: PMC3738824.

4. Ségurel L, Wyman MJ, Przeworski M. Determinants of mutation rate variation in the human germline. Annu Rev Genomics Hum Genet. 2014;15:47-70. doi: 10.1146/annurev-genom-031714-125740. PubMed PMID: 25000986.

5. Nielsen CT, Skakkebaek NE, Richardson DW, Darling JA, Hunter WM, Jørgensen M, et al. Onset of the release of spermatozoa (spermarche) in boys in relation to age, testicular growth, pubic hair, and height. J Clin Endocrinol Metab. 1986;62(3):532-5. doi: 10.1210/jcem-62-3-532. PubMed PMID: 3944237.

6. Drost JB, Lee WR. Biological basis of germline mutation: comparisons of spontaneous germline mutation rates among drosophila, mouse, and human. Environmental and molecular mutagenesis. 1995;25 Suppl 26:48-64. PubMed PMID: 7789362.

7. Gao JJ, Pan XR, Hu J, Ma L, Wu JM, Shao YL, et al. Pattern of mutation rates in the germline of Drosophila melanogaster males from a large-scale mutation screening experiment. G3 (Bethesda). 2014;4(8):1503-14. Epub 2014/06/14. doi: 10.1534/g3.114.011056. PubMed PMID: 24924332; PubMed Central PMCID: PMC4132180.

8. Gao JJ, Pan XR, Hu J, Ma L, Wu JM, Shao YL, et al. Highly variable recessive lethal or nearly lethal mutation rates during germ-line development of male Drosophila melanogaster. Proceedings of the National Academy of Sciences of the United States of America. 2011;108(38):15914-9. Epub 2011/09/06. doi: 10.1073/pnas.1100233108. PubMed PMID: 21890796; PubMed Central PMCID: PMC3179084.

9. Heller CG, Clermont Y. Spermatogenesis in man: an estimate of its duration. Science. 1963;140(3563):184-6. PubMed PMID: 13953583.

10. Behringer V, Deschner T, Deimel C, Stevens JM, Hohmann G. Age-related changes in urinary testosterone levels suggest differences in puberty onset and divergent life history strategies in bonobos and chimpanzees. Horm Behav. 2014;66(3):525-33. doi: 10.1016/j.yhbeh.2014.07.011. PubMed PMID: 25086337.

11. Smithwick EB, Young LG, Gould KG. Duration of spermatogenesis and relative frequency of each stage in the seminiferous epithelial cycle of the chimpanzee. Tissue & cell. 1996;28(3):357-66. Epub 1996/06/01. PubMed PMID: 8701437.
