# Supplementary material for: Interpreting the Dependence of Mutation Rates on Age and Time
Source: PLoS Biol. 2016 Jan 13;14(1):e1002355. doi: 10.1371/journal.pbio.1002355 (PMC4711947; doi:10.1371/journal.pbio.1002355)
Supplement: S2 Text — (DOC) [file pbio.1002355.s005.doc]

The analyses in the main text rely on several simplifying assumptions about the DNA damage, repair and replication processes. Here, we briefly discuss how the basic model can be extended to model more complex scenarios of mutagenesis and to understand more detailed patterns of mutations, such as the strand bias in mutation rates associated with transcription [1, 2]. Importantly, the analyses show that key qualitative behaviors of the basic model presented above and in the main text are robust to these extensions.

***1. Other miscoding lesions***

Although the basic model was presented as describing deamination of 5mC at CpG, it also applies to other types of miscoding lesions. If a miscoding lesion is not repaired correctly before DNA replication, it leads to the incorporation of an incorrect base in the newly synthesized strand. This results in a “semi-substitution” of the base pair after cell division. Although afterwards, the modification itself may still be recognizable by the DNA repair pathway, it is unlikely to be repaired correctly, because the original template is lost and substituted by another regular DNA base. Therefore, unrepaired miscoding lesions will almost always lead to mutations, just as in the case of deamination of 5-methylcytosine. Hence, our model can be applied to other miscoding lesions, such as O-6-ethylguanine and 8-oxoguanine that result from alkylation and oxidation of guanine, respectively [3], and the accrual of mutations will have the same behavior as described above (Fig 4B,C).

***2. Noncoding lesions***

The model can also describe the accumulation of mutations that result from noncoding lesions, i.e., abasic sites or modified DNA bases that cannot pair with any regular nucleotides. When the DNA replication machinery encounters such a lesion, it will either stall and then trigger cell death or bypass the lesion by cleaving the “irregular” base and incorporating a random base instead. In the latter case, the incorporated base is likely to differ from the original one and will result in a mutation in one of the two daughter cells. The probability that the randomly incorporated base happens to be the original one will depend on the concentrations of the four dNTPs in the cell, so the per cell division mutation rate is:

*M*_NR_(*T*)= ½ *Kp*1(*T*), (11)

where K (0<*K*<1) is a scale factor depending on the specific type of noncoding lesion and the concentrations of the four dNTPs in the cell. Therefore, *M_NR_*(*T*) has similar limiting behaviors as equation (5) (i.e., the linear phase and asymptotic phase).

There is one complication under this scenario though: because noncoding lesions can trigger cell death, the surviving cell lineages will carry fewer mutations on average than the expectation in equation (11). As a result, our model for miscoding lesions will tend to over-estimate the mutation rate for noncoding lesions; however, there should still exist two limiting phases. The linear phase is approximately the same as described in (11), because cell death is rarely triggered when a cell carries very few lesions [4]. The asymptotic phase will have a lower limit value though, as cells that carry greater numbers of noncoding lesions will be more likely to enter cell death.

***3. Different repair rates for the transcribed and non-transcribed strands***

Some types of mutations display substantial asymmetry between the transcribed and non-transcribed (coding) strands, as observed in both phylogenetic and cancer studies. In sequence divergence between humans and chimpanzees, A→G transitions on the transcribed strand are 58% more common than the complementary T→C transitions [1]. This phenomenon can be explained by transcription associated mutagenesis (TAM) [5] or by transcription-coupled repair (TCR), a sub-pathway of nucleotide excision repair (NER) that corrects errors on the transcribed strand of actively expressed genes [6]. In lung adenocarcinoma, G→T mutations, a signature associated with tobacco carcinogens, are found to be less prevalent on the transcribed strand; in turn, in malignant melanoma, C→T mutations, which likely result from exposure to UV radiation, also occur less often on the transcribed strand [2, 7]. These observations are both consistent with TCR.

Our model can be extended to incorporate TCR or TAM by modeling the accumulation of mutations on the two strands separately, where each strand has its own damage and repair rates. Notably, because damage rates are extremely small, we can assume that each strand experiences lesions independently, so the total number of mutations is the sum of unrepaired lesions on both strands by cell division. Assuming independence between the two strands, the results about the relationship between the efficiency of repair and the accumulation of mutations apply to both strands separately.

The model can then be used to infer parameters of interest, such as the relative difference in repair rates between the transcribed and non-transcribed strands. As an illustration, we assume that all or most mutations are non-replicative and that the strand bias is completely attributable to TCR. Assuming that the two strands have the same damage rate but different relative repair rates *R*_1_ and *R*_2_ (*R*_1_ > *R*_2_>0), the per cell division mutation rates for the two strands are:

$M_{NR,1}(T)=\frac{1}{2(1+R_{1})}(1-e^{-(1+R_{1})\mu T})$, and$M_{NR,2}(T)=\frac{1}{2(1+R_{2})}(1-e^{-(1+R_{2})\mu T})$.

If the fraction of mutations that accrue on the transcribed strand is x (*x*< ½), then:

$\frac{x}{1-x}=\frac{M_{1}(T)}{M_{2}(T)}=\frac{1+R_{2}}{1+R_{1}}\cdot\frac{1-e^{-(1+R_{1})\mu T}}{1-e^{-(1+R_{2})\mu T}}>\frac{1+R_{2}}{1+R_{1}}>\frac{R_{2}}{R_{1}}$,

which can be reorganized as:

$\frac{R_{1}}{R_{2}}>\frac{x}{1-x}$. (12)

The right-hand term of (12) provides a good approximation for the ratio of *R_1_* to *R_2_* when the repair rates are much greater than the damage rate (*R*_1_ > *R*_2_>>1) and repair is efficient on both strands (i.e., $(1+R_{2})\mu T$ is moderately large). Thus, in principle, the model could be used to make inferences about the relative rates of TCR.

***4. Repair with errors***

To take into account the repair machinery’s occasional failure to resolve mismatches correctly, we can include a repair error rate, *ɛ* (*ɛ*>0) in the model and assume that, when the repair is incorrect, it alters the strand without damage and leads to a base pair replacement. In this model, each DNA base pair can therefore be in one of three states: the original state, a state in which one strand has a lesion and one in which both strands changed (i.e., a base pair replacement). We denote the three states by S0, S1 and S2 respectively, and the proportions of base pairs in the states at time *t* since last cell division by *p*_0_(*t*), *p*_1_(*t*) and *p*_2_(*t*) (S1 Fig A). Therefore, the initial conditions are *p*_0_(0)=1 and *p*_1_(0)= *p*_2_(0)=0, and the dynamics of the three proportions follow the differential equations:

$\frac{dp_{0}}{dt}=-\mu p_{0}+(1-\varepsilon)rp_{1}$,

$\frac{dp_{1}}{dt}=\mu p_{0}-rp_{1}$,

$\frac{dp_{2}}{dt}=\varepsilon rp_{1}$.

where *p*_0_(*t*) + *p*_1_(*t*) + *p*_2_(*t*) = 1. If a base pair is in state S2 at the time of cell division, it leads to a base pair substitution in each of the two daughter cells. Therefore, the effective mutation rate per division is *M_NR_*(*T*)= ½ *p*_1_(*T*) + *p*_2_(*T*).

The solution to this differential equation system is:

, (13)

where *λ*_1_ and *λ*_2_ are the two eigenvalues of the matrix $\left[\begin{matrix} -\mu& (1-\varepsilon)r \\ \mu& -r \end{matrix}\right]$, and$\vec{\eta}_{1}$ and$\vec{\eta}_{2}$ are the corresponding vectors that satisfy , because of the initial condition.

It can be shown that:

$\lambda_{1}+\lambda_{2}=-(\mu+r)$;

$\lambda_{1}\lambda_{2}=\varepsilon\mu r$.

Therefore, *λ*_1_ and *λ*_2_ are both negative numbers, and under the assumption that *ɛ* is small, one of the two eigenvalues is very close to zero, and the other close to –(*μ*+*r*). Without loss of generality, let us assume that *λ_1_* is the one close to zero. Because of the similarity in form between equations (13) and (5), we predict that, when the error rate of repair is small, the results will be qualitatively similar to what we obtained from the simpler model. Indeed, we find that, for various values of *ɛ* (*ɛ* =0.0001, 0.001, 0.01 or 0.1), the behaviors of *p*_0_(*t*), *p*_1_(*t*) and *M_NR_*(*T*) at short time scales are very similar to the case with perfect repair (S1 Fig B). However, when *ɛ*>0, errors during repair will lead to accumulation of sites in state S2, and *M_NR_*(*T*) will eventually approach 1 when *T* is sufficiently large. In summary, the mutation accumulation curve can be roughly divided into four phases (see S1 Fig C):

Phase 1 (the linear phase): When (or approximately (*μ*+*r*)*t*<<1), *p*_1_(*t*) increases approximately linearly and is the main contributor to *M_NR_*(*T*)= ½ *p*_1_(*T*) + *p*_2_(*T*), because there are too few base pairs in state S2 to contribute to the mutation rate. The specific value of *ɛ* does not significantly affect the length of phase 1 and the behaviors of *p*_0_(*t*), *p*_1_(*t*) and *M_NR_*(*T*) in this phase. Therefore, equation (6) is a good approximation: *M_NR_*(*T*) increases linearly with *T*, and the mutation rate per unit of time is independent of cell division rate.

Phase 2 (the asymptotic phase): When (or approximately 1/(*μ*+*r*)<<*t*<<1/*ɛr*), *p*_1_(*t*) reaches an asymptote, and the contribution of *p*_2_(*t*) to the mutation rate is still very small compared to that of *p*_1_(*t*). Therefore, equation (7) is a good approximation for this phase, which means that *M_NR_*(*T*) is relatively constant irrespective of *T*, and the mutation rate per unit of time is proportional to the cell division rate. The existence of *ɛ* does not impact the behaviors of *p*_0_(*t*), *p*_1_(*t*) and *M_NR_*(*T*) in this phase, but the value of *ɛ* determines the length of this phase: smaller *ɛ* leads to a longer asymptotic phase due to later onset of the next phase. When the error rate is zero, we got infinitely long asymptotic phase (discussed in the main text); when the error rate approaches 1, the accumulation of mutations approaches linear (similar to the case with no repair but the mutation rate is doubled, because completely erroneous repair turns each single strand lesion into a mutation).

Phase 3 (the takeoff phase): when$|\lambda_{1}|t<<1<<\varepsilon rt$, *p*_1_(t) is still relatively constant with *t*, but the linear increase in *p*_2_(*t*) makes lesions in state S2 the main source of mutations in this phase. Therefore, *M_NR_*(*T*) takes off from the temporary asymptote in phase 2 and increases linearly with *t* again. Because$|\lambda_{1}|=\frac{\varepsilon\mu r}{|\lambda_{2}|}\approx\frac{\varepsilon\mu r}{\mu+r}$, the two boundaries of phase 3 are both proportional to ɛ, so the logarithm of its length is relatively constant. In this phase, *M_NR_*(*T*) increases linearly with *T* but is not proportional to *T*, so the mutation rate per unit of time depends on the cell division rate but not in strict proportion.

Phase 4 (the final phase): when, both *p*_0_(*t*) and *p*_1_(*t*) quickly drop to 0, and most base pairs in the genome are substituted (in state S2). Therefore, the mutation rate is approximately 1 in this phase. Although this regime is mathematically possible, it is almost certainly biologically irrelevant, given that the mutation rate per cell division is very low.

In summary, other than in the linear phase where repair is highly inefficient (i.e., (*μ*+*r*)*t*<<1), the rate of accrual of mutations per unit time will depend on the cell division rate. Thus, our key points highlighted the main text still hold even when the error rate is not zero but reasonably small (*ɛ*<0.1).

***5. Varying damage rate and/or repair rate***

Although we assume that the damage and repair rates are constant throughout time, in reality, they may vary considerably due to changes in chromatin state (e.g., the CpG methylation level [8]), concentrations of chemicals, and changes in expression levels of repair complex components with age or even within a cell cycle. To take into account changes in damage and repair rates with age, we can calculate the expected mutation rate by treating the accumulation of mutations as a piece-wise linear process and summing across all ages to obtain the mutation rate at any given age. To deal with varying parameters within a cell cycle, we can rewrite the differential equations by treating the damage and repair rates as functions of time since last cell division:

$\frac{dp_{0}}{dt}=-\mu(t)p_{0}+r(t)p_{1}$,

$\frac{dp_{1}}{dt}=\mu(t)p_{0}-r(t)p_{1}$,

where *μ*(*t*) and *r*(*t*) are known functions.

The differential equation can be solved as:

$p_{1}(t)=\frac{\int e^{U(t)+R(t)}\mu(t)dt-\frac{\mu(0)}{\mu(0)+r(0)}}{e^{U(t)+R(t)}}$, (14)

where$U(t)=\int_{0}^{t} \mu(t )dt$, and$R(t)=\int_{0}^{t} r(t )dt$. The mutation rate per cell division is *M_NR_*(*T*)= ½ *p*_1_(*T*).

If *μ*(*t*) and *r*(*t*) are well characterized, the mutation rate can be solved numerically using equation (14). Whether there exists a linear phase or an asymptotic phase depends on the specific forms of *μ*(*t*) and *r*(*t*). Nonetheless, *M_NR_*(*T*)/T is unlikely to be independent of *T*, so the mutation rate per unit time will likely be affected the cell division rate.

**References**

1. Green P, Ewing B, Miller W, Thomas PJ, Green ED, Program NCS. Transcription-associated mutational asymmetry in mammalian evolution. Nat Genet. 2003;33(4):514-7. doi: 10.1038/ng1103. PubMed PMID: 12612582.

2. Pleasance ED, Cheetham RK, Stephens PJ, McBride DJ, Humphray SJ, Greenman CD, et al. A comprehensive catalogue of somatic mutations from a human cancer genome. Nature. 2010;463(7278):191-6. Epub 2009/12/18. doi: 10.1038/nature08658. PubMed PMID: 20016485; PubMed Central PMCID: PMC3145108.

3. Friedberg EC. DNA repair and mutagenesis. 2nd ed. Washington, D.C.: ASM Press; 2006. xxix, 1118 p., [11] p. of plates p.

4. Otterlei M, Kavli B, Standal R, Skjelbred C, Bharati S, Krokan HE. Repair of chromosomal abasic sites in vivo involves at least three different repair pathways. EMBO J. 2000;19(20):5542-51. doi: 10.1093/emboj/19.20.5542. PubMed PMID: 11032821; PubMed Central PMCID: PMCPMC314018.

5. Jinks-Robertson S, Bhagwat AS. Transcription-associated mutagenesis. Annu Rev Genet. 2014;48:341-59. doi: 10.1146/annurev-genet-120213-092015. PubMed PMID: 25251854.

6. Hanawalt PC, Spivak G. Transcription-coupled DNA repair: two decades of progress and surprises. Nature reviews Molecular cell biology. 2008;9(12):958-70. Epub 2008/11/22. doi: 10.1038/nrm2549. PubMed PMID: 19023283.

7. Alexandrov LB, Nik-Zainal S, Wedge DC, Aparicio SA, Behjati S, Biankin AV, et al. Signatures of mutational processes in human cancer. Nature. 2013;500(7463):415-21. Epub 2013/08/16. doi: 10.1038/nature12477. PubMed PMID: 23945592; PubMed Central PMCID: PMC3776390.

8. Horvath S. DNA methylation age of human tissues and cell types. Genome Biol. 2013;14(10):R115. doi: 10.1186/gb-2013-14-10-r115. PubMed PMID: 24138928; PubMed Central PMCID: PMCPMC4015143.
